# Case Report: Successful primary sutureless repair of common pulmonary vein atresia in a neonate

**DOI:** 10.3389/fped.2026.1895693

**Published:** 2026-08-20

**Authors:** Zhaoyang Zheng, Jinmei Zhang

**Affiliations:** Department of Pediatric Cardiovascular Surgery, West China Second University Hospital, Sichuan University, Chengdu, China

**Keywords:** cardiac surgery, case report, common pulmonary vein atresia, extracorporeal membrane oxygenation, sutureless anastomosis

## Abstract

Common pulmonary vein atresia (CPVA) is an extremely rare and lethal congenital heart defect characterized by the absence of a functional connection between the common pulmonary vein chamber (CPVC) and both the left atrium and systemic venous system. We report a case of a full-term female infant who successfully underwent primary sutureless repair. The patient developed acute respiratory distress syndrome (ARDS) with severe cyanosis and refractory hypoxemia 12 min after birth and underwent emergent surgery at 15 h of life. Intraoperative findings confirmed CPVA with bilateral superior pulmonary vein stenosis. Sutureless anastomosis was performed, leaving a 2.5 mm interatrial communication. Due to severe pulmonary hypertension and high-dose vasoactive requirements, venoarterial extracorporeal membrane oxygenation (VA-ECMO) support was established immediately. ECMO was weaned on postoperative day 6, the patient was extubated on day 10, and discharged on day 43. Two-month follow-up showed unobstructed pulmonary venous flow without evidence of anastomotic stenosis. To our knowledge, this is the first report of successful primary sutureless repair of this cardiac malformation.

## Introduction

First described by Lucas et al. (1) in 1962, common pulmonary vein atresia (CPVA) is recognized as the common pulmonary vein chamber (CPVC) that has no functional communication with the left atrium or any other cardiac chamber or systemic venous structure, resulting in complete obstruction to the pulmonary venous return. Approximately 45 cases have been reported, of which 13 underwent surgery, with only 7 successful survivors. The mortality rate is 100% without surgical intervention (2–7).

Infants with CPVA present with severe respiratory distress, refractory hypoxemia, and metabolic acidosis immediately after birth, with rapid disease progression. Clinical manifestations are similar to those of total anomalous pulmonary venous connection (TAPVC), and the diagnostic confirmation by echocardiography is often limited by poor acoustic window, leading to delayed treatment (4, 5).

Surgical repair is the only curative treatment. The sutureless technique can help avoid damaging the pulmonary vein wall and theoretically reduces the risk of anastomotic obstruction (8), but its application in CPVA has rarely been reported (7). We report the first case of successful surgical repair using primary sutureless technique.

## Case report

The patient was a full-term baby girl delivered by cesarean section with a birth weight of 3,920 g. She developed cyanosis of the face and lips 12 min after birth. Despite positive pressure ventilation at the outside hospital, oxygen saturation remained only 75%–80%. The neonate was transferred to our hospital 24 min after birth. Her heart rate was 165 beats/min, blood pressure was 40/30 mmHg, and SpO_2_ was 65.6% at admission. Arterial blood gas analysis revealed severe acidosis (pH 7.144), hypercapnia (PaCO_2_ 67.0 mmHg), hypoxemia (PaO_2_ 35.1 mmHg), and lactate 1.6 mmol/L.

**Figure 1:**
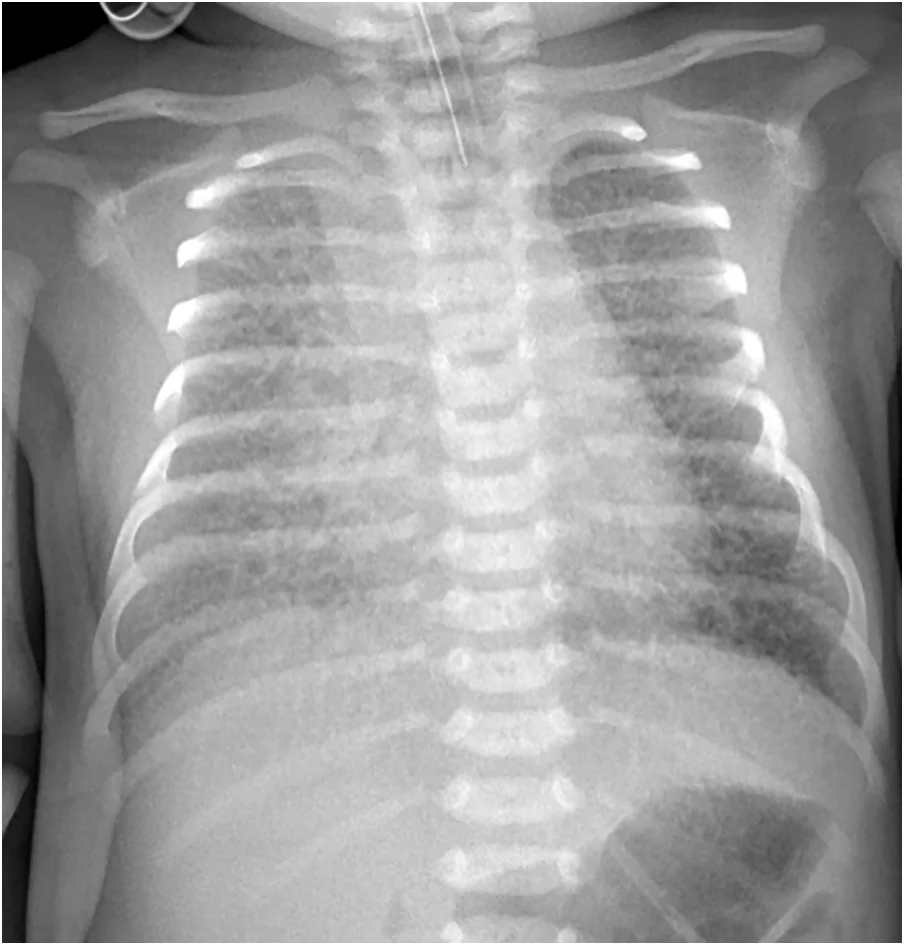
Chest x-ray soon after birth in a neonate with CPVA shows indistinct vascular markings, reduced pulmonary translucency, and normal cardiac silhouette. *CPVA*, common pulmonary vein atresia.

**Figure 2:**
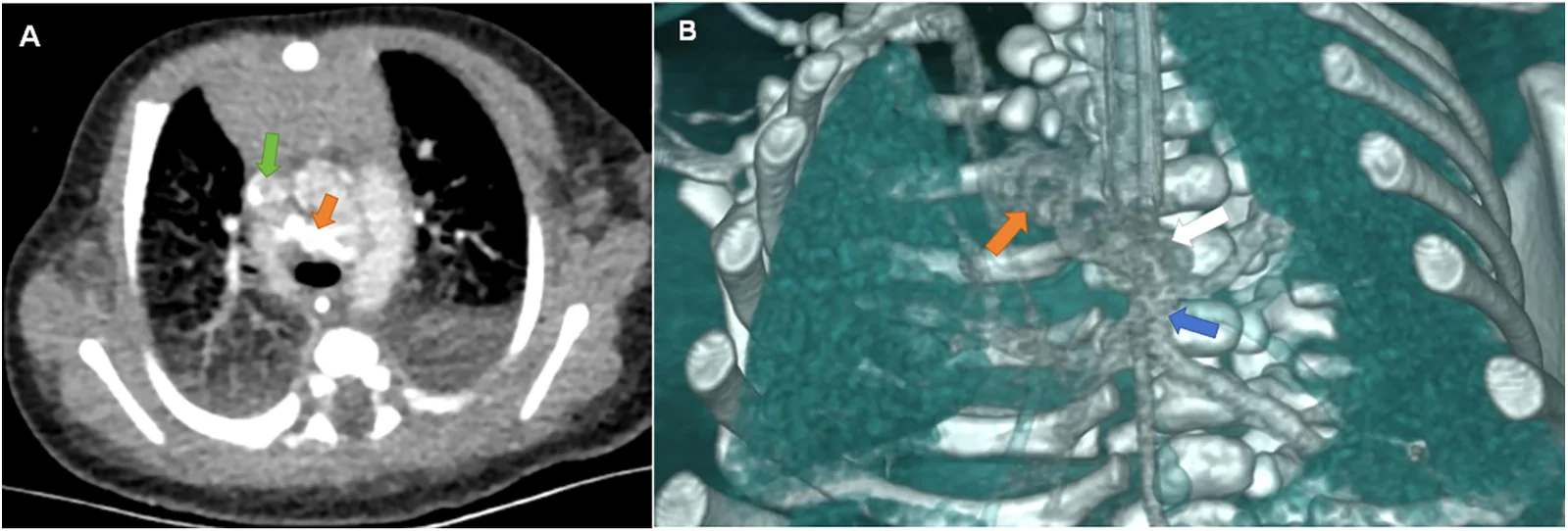
Cardiac CTA **(A)** with three-dimensional reconstruction **(B)** demonstrates a CPVC (blue arrow) and ascending vertical vein (white arrow), forming a tortuous vascular mass (orange arrow) anterior to the trachea and posterior to the SVC (green arrow) in the patient. *CTA*, computed tomographic angiography; *CPVC*, common pulmonary venous chamber; *SVC*, superior vena cava.

Chest x-ray showed diffuse bilateral reticulogranular infiltrates with patchy opacities and reduced pulmonary translucency, consistent with neonatal pneumonia and acute respiratory distress syndrome (ARDS), with a normal cardiac silhouette (Figure 1). Echocardiography revealed right ventricular enlargement (12 mm), a 15.3 mm atrial septal defect (ASD) with predominant right-to-left shunt, a 3.3 mm patent ductus arteriosus (PDA), visualization of only two pulmonary veins entering the left atrium, and reversed flow in the aortic arch. Cardiac computed tomographic angiography (CTA) demonstrated that the four pulmonary veins converged into a common venous chamber, forming a tortuous vascular mass anterior to the trachea and posterior to the superior vena cava (SVC) (Figure 2), with an indistinct drainage pathway.

**Figure 3:**
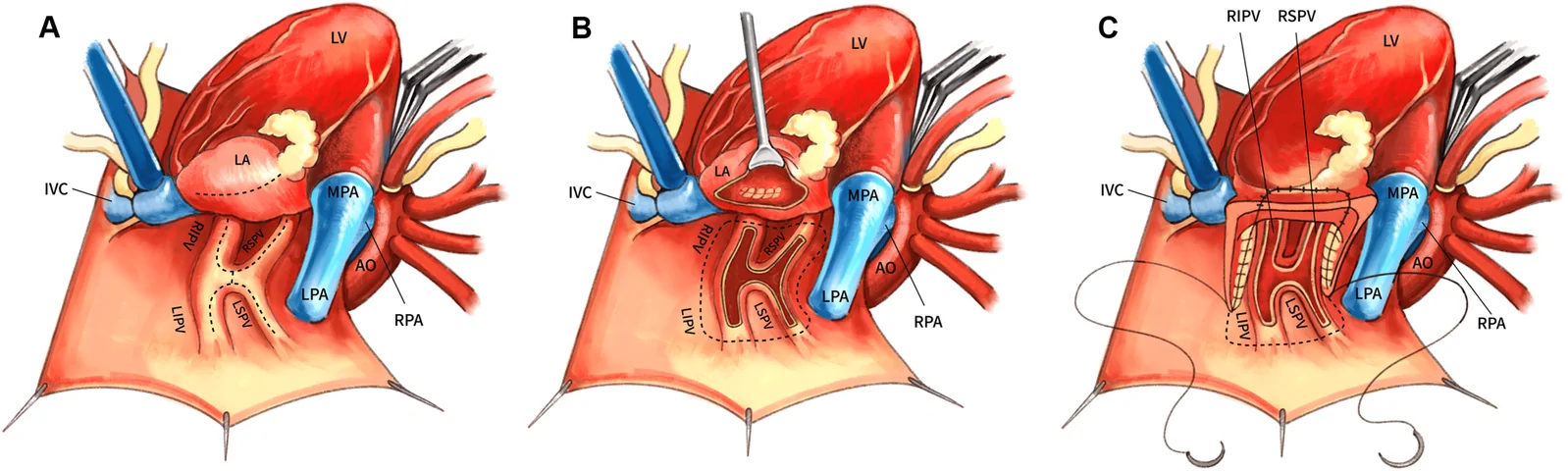
Schematic illustration of the sutureless technique for repair of CPVA. **(A)** A transverse incision was made across the CPVC, and a longitudinal incision was performed along the left atrial appendage. **(B, C)** The pericardial tissues surrounding the CPVC and left atrium were anastomosed to create a wide, tension-free opening without direct suturing of the pulmonary vein. *AO*, aorta; *CPVA*, common pulmonary vein atresia; *CPVC*, common pulmonary venous chamber; *IVC*, inferior vena cava; *LA*, left atrium; *LIPV*, left inferior pulmonary vein; *LPA,* left pulmonary artery; *LSPV*, left superior pulmonary vein; *LV*, left ventricle; *MPA*, main pulmonary artery; *RIPV*, right inferior pulmonary vein; *RPA*, right pulmonary artery; *RSPV*, right superior pulmonary vein.

**Figure 4:**
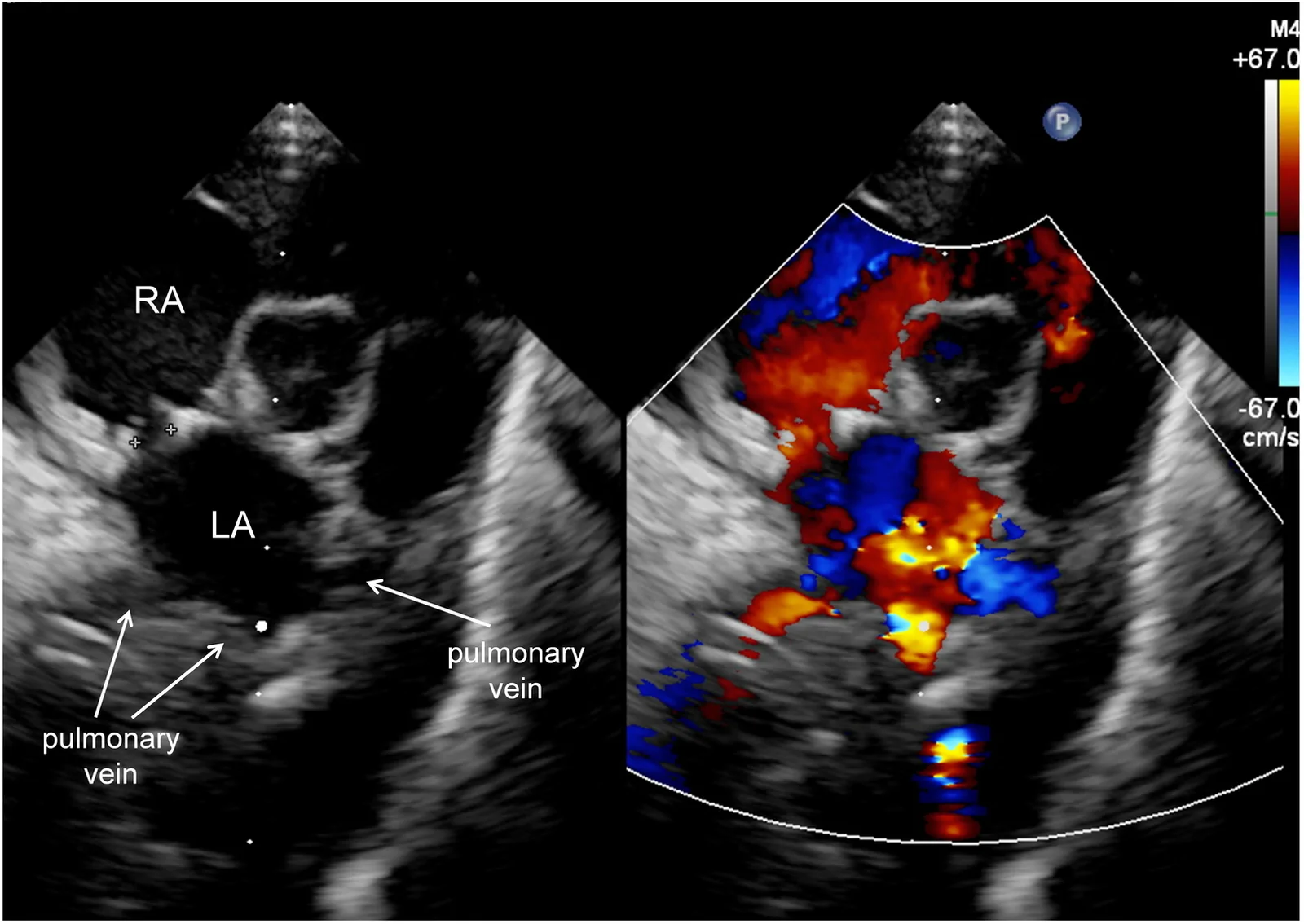
Two-dimensional echocardiography in the immediate postoperative period after sutureless repair of CPVA shows unobstructed pulmonary venous drainage into the left atrium. *CPVA*, common pulmonary vein atresia; *RA*, right atrium; *LA*, left atrium.

Given the rapidly deteriorating condition, the infant underwent emergent surgery at 15 h of life. A midsternal thoracotomy was performed to expose the heart and the PDA was ligated. A dilated and tortuous venous plexus was observed adjacent to the anterior wall of the ascending aorta. The four pulmonary veins converged behind the left atrium, with no vertical vein identified. Both superior pulmonary veins were severely dysplastic (diameter approximately 1 mm), while the inferior pulmonary veins were relatively well developed. Cardiopulmonary bypass (CPB) was established via the ascending aorta and bicaval cannulation. After cross-clamping the ascending aorta, HTK (histidine-tryptophan-ketoglutarate) crystalloid cardioplegia (30–50 mL/kg) was administered to induce cardiac arrest. A transverse incision was made across the CPVC, reaching the orifices of the left and right pulmonary venous branches. A longitudinal incision was performed along the midline of the left atrial appendage and extended to the posterior wall of the left atrium. The pericardium around the common trunk of the pulmonary vein and the left atrium was then anastomosed using a sutureless technique to create a wide opening. The key steps of the sutureless anastomosis is illustrated in Figure 3. The ASD was subsequently closed, leaving a small residual interatrial communication (2.5 mm). Postoperative echocardiography showed all pulmonary veins draining into the left atrium (Figure 4), and mild tricuspid regurgitation. Doppler velocities were as follows: right superior pulmonary vein, 0.9 m/s; right inferior, 0.65 m/s; left superior, 1.1 m/s; and left inferior, 0.8 m/s. The operation lasted 335 min, with CPB time of 117 min and aortic cross-clamping time of 63 min.

The patient could not be weaned from CPB due to severe pulmonary hypertension (left ventricular systolic pressure to right ventricular systolic pressure ratio of 79:66 mmHg), high-dose vasoactive support (vasoactive-inotropic score 56), and elevated lactate (6.0 mmol/L). Therefore, venoarterial extracorporeal membrane oxygenation (VA-ECMO) support was initiated immediately in the operating room, along with delayed sternal closure.

The infant was then transferred to the cardiac intensive care unit (CCU) for continued management. ECMO was successfully weaned off on postoperative day 6, the chest was closed on day 7, and the tracheal tube was removed on day 10. The patient was discharged on day 43 with a weight of 4,760 g, showing no cyanosis or shortness of breath without oxygen inhalation. Two-month follow-up echocardiography showed left ventricular ejection fraction of 73%, unobstructed flow in all four pulmonary veins with maximum velocity of 1.5–1.6 m/s, residual ASD of 3.5 mm, and no evidence of anastomotic stenosis.

## Discussion

### Embryological considerations and diagnostic challenge

The embryological basis of CPVA lies in the failure of connection between the CPVC and the developing left atrium during late embryogenesis. Under such circumstances, pulmonary venous blood returns only through microscopic collateral channels, creating a phenotype of “absence of a functional connection” (1, 9). This embryological feature determines the diagnostic challenges of CPVA. Prenatal ultrasound often misses detection or misdiagnoses it as TAPVC due to low pulmonary blood flow during fetal life. Key postnatal diagnostic clues on echocardiography include the inability to trace a clear path of venous drainage and reverse flow in the pulmonary artery and aortic isthmus (10). Cardiac CTA is valuable for preoperative evaluation by providing detailed three-dimensional anatomical delineation of the CPVC and its spatial relationship with adjacent structures, particularly in fragile neonates where cardiac catheterization may be poorly tolerated (11).

### Timing of surgery and surgical strategy

The timing of surgical intervention is an important prognostic factor. In previously reported successful cases, surgery was performed within 16 h after birth (3, 12, 13). Delayed surgical timing often leads to unpredictable serious consequences. Nakamura et al. (4) reported a case where surgery was performed at 13 h of birth, but severe preoperative acidosis (lactate 173 mg/dL) led to brain damage and death at postoperative day 24. Zhang et al. (7) reported surgery at 22 h of birth, but the optimal window had been missed, and the patient died of multi-organ failures despite ECMO support. Our patient underwent surgery at 15 h of life. Intraoperative findings confirmed severe dysplasia of both superior pulmonary veins, which might have resulted in loss of surgical opportunity if delayed. We therefore recommend surgery within 24 h or even earlier after birth, as the delay of surgery may cause deterioration and death or perhaps the requirement for ECMO support (13).

The anastomosis technique has evolved over time. Conventional surgery anastomoses the pulmonary veins with the CPVC directly (3, 4, 12–16), which carries risk of postoperative pulmonary vein obstruction (PVO) due to intimal hyperplasia caused by suture irritation. The sutureless technique, which is associated with significantly lower rates of PVO in TAPVC repair (8), has been rarely reported in CPVA. Zhang et al. (7) described a case of sutureless repair. Severe fibrous tissue hyperplasia developed at the anastomotic site and extended to the pulmonary venous orifice. Reoperation was required 52 days later; however the child ultimately failed to wean from CPB. In this case, we adopted sutureless technique with interatrial shunt preservation based on three technical considerations. First, the transverse incision across the CPVC was extended to the orifices of both pulmonary venous branches to create a wide anastomotic opening. Second, the anastomosis between the left atrium and the pericardial tissue surrounding the CPVC served to expand the opening. Importantly, this technique also avoided direct suture irritation of the pulmonary vein intima. Third, a 2.5 mm interatrial communication was left as a right-heart decompression strategy during the early postoperative period of pulmonary hypertension (14). Postoperative echocardiography at 2 months showed unobstructed pulmonary venous flow with maximum velocity of 1.6 m/s, preliminarily verifying the effectiveness of this technique.

### Postoperative management and follow-up

Postoperative pulmonary hypertension is almost universal in CPVA and often requires nitric oxide support or extracorporeal life support (4, 7, 13). Pulmonary lymphangiectasia (PL) is another common complication associated with severe PVO, potentially affecting long-term prognosis (7, 13). Mas et al. (13) reported a patient who received immediate postoperative iNO and supportive measures for PL-related complications. Respiratory and myocardial functions recovered after 7 weeks of mechanical ventilation. In a 1993 case, postoperative ECMO support provided sufficient recovery time for pulmonary hypertension and severe pulmonary parenchymal disease (17). In our case, VA-ECMO was established immediately after the procedure, gaining time for cardiopulmonary recovery. ECMO was successfully weaned off on postoperative day 6, and the ventilator was removed on day 10. The duration of ventilator support was significantly shorter than the 7 weeks reported by Mas et al. (13). Notably, no chylothorax or air leaks occurred, possibly due to careful intraoperative inspection and early fluid restriction with diuresis.

Long-term follow-up data for CPVA are limited. Successful cases have been followed from 4 months (3) to 5 years (12) without evidence of pulmonary hypertension or re-obstruction. Re-obstruction may occur months to years later due to anastomotic fibrous hyperplasia (7). Our 2-month follow-up showed unobstructed pulmonary venous drainage, preserved cardiac function (ejection fraction 73%), and no anastomotic stenosis. However, close long-term follow-up is indispensable, with cardiac CTA additionally performed to assess the patency at the anastomotic sites if necessary.

## Conclusion

CPVA is a life-threatening and rare cardiac malformation in the neonatal period. Early recognition, emergent surgery within 24 h, delicate sutureless anastomosis technique, and active postoperative cardiopulmonary support are key to improving prognosis. Our successful experience offers insight into feasible therapeutic strategies in this rapidly fatal disease.

## Data Availability

The datasets presented in this article are not readily available because of ethical and privacy restrictions. Requests to access the datasets should be directed to the corresponding author/s.
